# Symptomatic Intracranial Hemorrhage after Ischemic Stroke Treated with Bridging Revascularization Therapy

**DOI:** 10.3390/life13071593

**Published:** 2023-07-20

**Authors:** Simon Amaral, Gauthier Duloquin, Yannick Béjot

**Affiliations:** 1 Neurology Department, Dijon University Hospital, 21000 Dijon, France; 2 Dijon Stroke Registry, EA7460, University of Burgundy, 21078 Dijon, France

**Keywords:** ischemic stroke, IV thrombolysis, mechanical thrombectomy, bridging therapy, intracranial hemorrhage, outcome

## Abstract

(1) Background: bridging revascularization therapy is now the standard of care in patients with ischemic stroke due to large vessel occlusion. This study aimed to determine the frequency of symptomatic intracranial hemorrhage (sICH) related to this treatment, and to assess contributing factors and patients’ outcomes. (2) Methods: consecutive ischemic stroke patients treated with bridging therapy were prospectively enrolled. sICH (intracranial hemorrhage with an increase in NIHSS score of ≥4 points) was assessed on imaging at 24 h. The functional status of patients was measured at 6 months using the mRS score; (3) Results: 176 patients were included (mean age 68.7 ± 1.2 years, 52.3% women), among whom 15 (8.5%) had sICH. Patients with sICH had more frequent alcohol abuse (30.1% versus 9.7%, *p* = 0.023), prestroke use of dual antiplatelet therapy (14.3% versus 1.3%, *p* = 0.002), higher NIHSS scores at admission (median score 20.5 versus 15, *p* = 0.01), greater systolic blood pressure upon admission, more frequent vascular intracranial calcifications (*p* = 0.004), leukoaraiosis (*p* = 0.001), and intracranial atheroma (*p* = 0.02), and higher neutrophil-to-lymphocyte ratios (*p* = 0.02) and neutrophil-to-platelet ratios (*p* = 0.04). At 6-month follow-up, 9 (60%) patients with sICH died, versus 18% of patients without sICH (*p* < 0.001). Only 1 (7%) patient with sICH had a good functional outcome, defined as an mRS score of 0 to 2, versus 51% of patients without sICH. (4) Conclusions: one in twelve ischemic stroke patients treated with bridging therapy suffered sICH. Given the observed poor outcomes after sICH, further studies are required to better identify patients at risk to help clinicians in guiding therapeutic strategies.

## 1. Introduction

Stroke is the second leading cause of death and disability worldwide, with more than 6 million deaths and 143 million disability-adjusted life years (DALYs) each year, and the aging population will lead to a dramatic rise in the burden of the disease in the coming years in both high- and low-to-middle-income countries [1,2]. Since the 1990s, intravenous thrombolysis (IVT) using recombinant tissue plasminogen activator (rt-PA) has been shown to reduce disability among patients suffering acute ischemic stroke [3]. After the publication of a series of randomized clinical trials in 2015 and beyond, mechanical thrombectomy (MT), in association with IVT, was demonstrated to be superior to IVT alone in improving outcomes after 3 months in patients with ischemic stroke due to a large vessel occlusion (LVO) of the anterior circulation [4,5,6,7,8,9], who account for approximately 17% of overall cases [10]. In these patients, the combination of IVT and MT, also called bridging therapy, is now recognized as the standard of care, according to current guidelines for the acute management of ischemic stroke [11].

The most common and dreaded complication of acute revascularization therapy is hemorrhagic transformation or symptomatic intracranial hemorrhage (sICH), the frequency of which was estimated at around 6–8% after IVT [12,13,14,15], and between 4% and 7% after MT [16], depending on the definition considered. sICH has a negative impact on clinical outcomes, with increased mortality and severe disability [15]. Several risk factors for sICH have been established for both IVT and MT, but there is less evidence regarding bridging therapy.

The aim of this study was to determine the frequency of sICH in ischemic stroke patients treated with bridging therapy, to identify contributing factors, and to assess patients’ outcomes at 6 months.

## 2. Materials and Methods

### 2.1. Study Population

This study was based on the Prognosis After Revascularisation therApy in the Dijon Ischemic Stroke Evaluation (PARADISE) Study. This was a monocentric prospective observational cohort study conducted between January 2016 and June 2019 at the stroke unit of the tertiary center of the University Hospital of Dijon, France (Clinical Trial NCT02856074). As previously described [17], all patients aged 18 years or greater who had a diagnosis of acute ischemic stroke confirmed by clinical presentation and brain imaging and who received a revascularization therapy, including either IVT alone, MT alone, or bridging therapy, were enrolled consecutively. Oral consent to participate was collected from the patients, or from their relatives in cases in which it was impossible for the patients to provide it (because of altered consciousness, severe cognitive impairment, or aphasia), according to the French legislation. The study was approved by a French ethics committee (Comité de Protection des Personnes CCP Est I, IRB number: 2015-A01664-45). During the study period, IVT was performed when indicated, according to the current guidelines on the acute management of ischemic stroke, with rt-PA at the standard dose of 0.9 mg/kg and without exceeding 90 mg. Patients were treated either locally at the stroke unit of Dijon University Hospital, or received IVT during a telemedecine procedure in a community hospital of the administrative region of Burgundy before being transported to the Dijon stroke unit, as part of the Burgundy Telestroke Network, as previously described [18]. MT was performed in patients with ischemic stroke and an LVO by a trained neuroradiologist of Dijon University Hospital, using an FDA-approved stent retriever. The anesthesia procedure was left to the neuroradiologist’s choice according to the patient’s clinical condition. For this current study, we extracted the data of patients who beneficiated from bridging therapy, i.e., the association of both IVT and MT.

### 2.2. Data Collection

Demographics, past medical history, vascular risk factors (arterial hypertension, diabetes mellitus, smoking, hypercholesterolemia, alcohol abuse, obesity, and atrial fibrillation) and premorbid treatments (antiplatelet agents, anticoagulants, antihypertensive therapy, antidiabetics, statin therapy, antidepressants, and antipsychotics) were recorded prospectively. Pre-stroke cognitive status was assessed based on interviews with patients and/or their relatives, and according to the medical files of patients. We considered no cognitive impairment versus mild cognitive impairment (MCI), defined as a cognitive decline without any interference with activities of daily life, and dementia, defined as cognitive decline sufficient to interfere with independence in the activities of daily living (according to the Diagnostic and Statistical Manual of Mental Disorders (DSM-V) criteria), or patients receiving specific treatments for Alzheimer’s disease. There was no formal cognitive testing at the acute stage of stroke; therefore, adjudication was made by a senior physician with expertise in both stroke and dementia, based on information collected. On the first clinical examination, the National Institutes of Health Stroke Scale (NIHSS) score was calculated by a stroke-trained neurologist in charge of the management of the patient, in order to evaluate stroke severity.

At hospital admission, all patients underwent either a brain CT scan or/and magnetic resonance imaging (MRI), according to local procedures. The occlusion site was assessed on an angio-CT scan or an angio-MRI by stroke neurologists and neuroradiologists. We distinguished occlusion of the anterior circulation, including the internal carotid artery (ICA), M1 or M1–M2 junction segment of the middle cerebral artery (MCA), M2 segment of the MCA, tandem occlusion, or occlusion of the terminal ICA, from occlusion of the posterior circulation (vertebral or basilar artery). Extracranial and intracranial atheroma was recorded based on the results of vascular imaging, including an additional US Doppler of cervical arteries performed during the diagnostic work-up. The presence of vascular calcifications was assessed according to the findings of the angio-CT-scan when available. We considered calcification of the internal carotid bulb, carotid siphon, and V4 segment of the vertebral arteries. In patients who had a brain MRI, leukoaraiosis was assessed on a fluid-attenuated inversion recovery (FLAIR) sequence, and microbleeds were searched for using a T2 gradient echo sequence. After diagnostic work-up, we defined cardioembolic stroke as ischemic stroke related to a major source of cardioembolism, including atrial fibrillation, left ventricular thrombus, left atrial thrombus, recent myocardial infarction, endocarditis, valve prosthesis dysfunction, and intracardiac tumor. Large artery atherosclerosis stroke was considered in cases of significant atheroma plaque affecting the artery involved in the ischemic stroke territory, according to the clinicians’ judgment.

All patients gave biological samples on admission to measure leukocyte and platelet counts, thyroid-stimulating hormone (TSH), C-reactive protein (CRP), hemoglobin A1C (HbA1c), creatinine, troponin Ic, and lipid levels (including high-density lipoprotein (HDL) cholesterol, low-density lipoprotein (LDL) cholesterol, and total cholesterol levels). The monocyte-to-HDL ratio, neutrophil-to-lymphocyte ratio, and neutrophil-to-platelet ratio were calculated, as these have been demonstrated to be indicators of systemic inflammation and oxidative stress. Time between stroke onset and both IVT (onset-to-needle time) and MT (onset-to-puncture time) were recorded.

Intracranial hemorrhage was systematically evaluated with a CT-scan or MRI performed 24 h after revascularization therapy, or before in case of a clinical neurological deterioration of the patient. We used the European Cooperative Acute Stroke Study (ECASS-II) definition to classify intracranial hemorrhage as follows: small petechiae along the margins of the infarcted area (HI-1), confluent petechiae within the infarct without mass effect (HI-2), hematoma occupying 30% or less of the infarcted area (PH-1), and hematoma in more than 30% of the infarcted area with mass effect (PH-2) [19]. sICH was considered as intracranial hemorrhage at any site of the brain associated with a neurological deterioration characterized by an increase in the NIHSS score of 4 or more points.

### 2.3. Follow-Up

Patients were followed up with a face-to-face visit at 6 months with a stroke-trained neurologist. The functional status of patients was systematically assessed with the modified Rankin scale (mRS) score, ranging from 0 (corresponding to patients with no disability) to 6 (corresponding to patients who died). A good functional outcome was defined as a mRS score of 0, 1 or 2, in accordance with the definition commonly used in most randomized clinical trials dealing with the acute treatment of ischemic stroke with IVT or MT. This corresponds to patients with no symptoms and those with a slight disability. For patients who did not attend the visit, information on the mRS score was evaluated with a structured questionnaire performed during a telephone interview with the patient, or with a relative or a caregiver if the patient was unable to answer the questions. In addition, for patients lost to follow-up, information on vital status was searched for using openly available national data on death certificates, which allowed for exhaustive information.

### 2.4. Statistical Analysis

Categorical variables were presented as frequencies and continuous variables as means ± standard deviations (SD) for normal distribution or medians (interquartile ranges, IQRs) for non-normal distribution. The normality hypothesis was verified graphically using histograms and statistically with the Shapiro–Wilk test. Data were presented for two groups of patients according to the occurrence of sICH. Comparisons between groups were made with a Mann–Whitney U test for continuous variables and the χ^2^ test or Fisher’s exact test for categorical variables. Statistical significance was assessed according to a two-tailed α level of 0.05. Statistical analysis was performed using Stata 13 software (StataCorp LP, College Station, TX, USA).

## 3. Results

**Figure 1:**
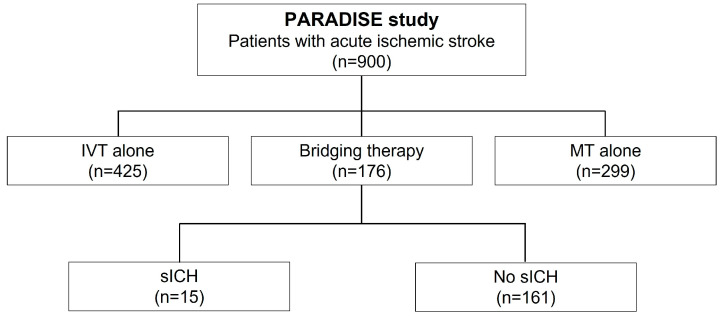
Study flowchart. PARADISE: Prognosis After Revascularisation therApy in the Dijon Ischemic Stroke Evaluation; IVT: intravenous thrombolysis; MT: mechanical thrombectomy; sICH: symptomatic intracerebral hemorrhage.

Among a total of 900 ischemic stroke patients prospectively included in the PARADISE study (mean age: 72.3 ± 14.5 years, 49.9% women, median NIHSS score 11, IQR: 6–18), 425 (47.2%) received IVT alone, 299 (33.2%) benefited from MT alone, and a total of 176 (19.6%) were treated with bridging therapy (Figure 1).

The mean age of ischemic stroke patients treated with bridging therapy was 68.7 ± 16.1 years old, and 52.3% of these patients were women. Among these patients, one hundred (56.9%) received IVT before MT in a community hospital of the administrative region of Burgundy during a telemedicine procedure, before being transferred to the University Hospital of Dijon for additional MT. The occlusion sites were distributed as follows: 8 (4.7%) patients had occlusion of the ICA, 32 (18.7%) patients had a tandem occlusion, 84 (49.1%) patients had occlusion of the M1 segment of the MCA, 14 (8.2%) patients had occlusion of the M1-M2 junction segment of the MCA, 12 (7%) patients had occlusion of the M2 segment of the MCA, 20 (11.7%) patients had occlusion of the basilar artery, and 1 (0.5%) patient had occlusion of a vertebral artery. The mean onset-to-needle time was 171.3 min, and the mean onset-to-puncture time was 308.6 min.

Some 15 (8.5%) patients had sICH after bridging therapy. The baseline characteristics of patients according to the occurrence of sICH are shown in Table 1. Patients with sICH had a greater prevalence of alcohol abuse than those without sICH (30.1% versus 9.7%, *p* = 0.023). There were no differences in demographics and other vascular risk factors or medical history between groups.

The distribution of prior-to-stroke treatment is shown in Table 2. Premorbid use of dual antiplatelet therapy (DAPT) was greater in ischemic stroke patients with sICH (14.3% versus 1.3%, *p* = 0.002). Of note, there was a non-significant trend toward a greater prevalence of antihypertensive therapy use among these patients (85.7% versus 62.5%, *p* = 0.08).

Regarding clinical features at onset (Table 3), ischemic stroke patients with sICH had a higher NIHSS score on admission (median NIHSS score 20.5 versus 15, *p* = 0.01) and more frequently altered consciousness (33.3% versus 11.4%, *p* = 0.01). In addition, a greater admission systolic blood pressure (SBP) was observed (median SBP: 175 mmHg versus 150.5 mmHg, *p* = 0.02; proportion of patients with a SBP on admission ≥ 160 mmHg: 77.7% versus 36.1%, *p* = 0.01). On imaging, patients with sICH differed from those without sICH regarding more frequent vascular intracranial calcifications (*p* = 0.004), leukoaraiosis on MRI (*p* = 0.001), and intracranial atheroma (*p* = 0.02) (Table 3). Conversely, patients did not differ regarding acute therapy procedures including onset-to-needle time, onset-to-groin puncture time, MT procedure duration, use of general anesthesia for MT, and rate of successful reperfusion (defined using the treatment in cerebral ischemia scale, TICI, 2b/3).

On biological sample, patients with sICH had higher neutrophil-to-lymphocyte ratios (*p* = 0.02) and the neutrophil-to-platelet ratios (*p* = 0.04) (Table 4) at admission, but did not differ with regard to other markers.

Information about 6-month functional outcomes was complete for 14 (93.3%) ischemic stroke patients with sICH, and for 145 (90%) patients without sICH. Information on vital status was complete for all patients included in this analysis. At 6 months, 9 (60%) patients with sICH died, versus 18% of patients without sICH (*p* < 0.001). Only 1 (7%) patient with sICH had a good functional outcome, defined as an mRS score of 0 to 2 (versus 51% of patients without sICH, *p* < 0.001).

## 4. Discussion

This study demonstrated that sICH defined according to the ECASS-II criteria occurred in 8.5% of ischemic stroke patients treated with bridging therapy, and was associated with a poor functional and vital outcome after 6 months of follow-up.

This rate of sICH was slightly higher than the 5–8% reported in pivotal randomized clinical trials that evaluated either IVT or MT [20]. A meta-analysis based on 4254 patients with acute ischemic stroke who received bridging therapy found a rate of hemorrhagic transformation of 6.8% [21]. In addition, a matched-control study that compared MT alone to bridging therapy in an Asian population found a 10% rate of sICH in the bridging therapy group [22]. The small differences between studies can be explained by discrepancies in the initial clinical severity of enrolled patients (in our study, the median NIHSS score was 15 (IQR: 9–20), which is considered as high), but also by the heterogeneity of the criteria used to define sICH in the literature, and the imaging modalities (plain brain CT scan versus MRI) used to diagnose it. We chose to consider the ECASS-II definition of sICH instead of considering any hemorrhagic transformation without taking into account change in the NIHSS score, because of its clinical relevance and ability to better reflect the neurological deterioration of patients, and its consequences for post-stroke outcomes.

In addition, recent randomized clinical trials that aimed to compare bridging therapy to MT alone in patients with acute ischemic stroke due to a large vessel occlusion reported rates of sICH in the bridging therapy group ranging from 1% to 11.7%, according to the definition considered (Table 5) [23,24,25,26,27,28]. The highest rates of sICH were observed when applying the overly inclusive NINDS definition, in which hemorrhagic transformation was considered symptomatic in cases of any neurological deterioration, without defining a threshold in terms of a worsening NIHSS score. In contrast, the low rate of sICH reported in the DIRECT-SAFE trial can be explained by the use of a very restrictive definition that considered only very severe intracerebral hemorrhage. ECASS-II, ECASS-III, SITS-MOST and Heidelberg classifications take into account a significant neurological deterioration to define sICH (i.e., an increase of ≥4 points in the baseline NIHSS score, or increase of ≥2 points in a NIHSS subcategory for Heidelberg classification). Therefore, when using these classifications, the actual rates of sICH ranged between 4.3% and 8.5%, which is consistent with the rate observed in our study.

Regarding contributing factors, alcohol abuse appeared as a risk factor that was more frequently observed among ischemic stroke patients with sICH in our study. This finding could be attributed to the deleterious effect of alcohol consumption on both the coagulation system and platelet function, which promotes the risk of bleeding. We did not collect results for coagulation tests performed routinely. However, the fact that patients received IV thrombolysis indicates that their tests were within the normal range, otherwise they would have been excluded. In addition, a retrospective review of the medical files of patients with sICH did not reveal coagulation abnormalities. Platelet levels also did not differ between patients with and patients without alcohol abuse. As a result, the deleterious effect of alcohol could involve qualitative rather than quantitative mechanisms. Stroke severity, as determined either by the NIHSS score or the presence of a decreased consciousness, was also greater in patients who developed sICH after bridging therapy than in their counterparts without sICH. As stroke severity on clinical examination often reflects the extent of the ischemic brain injury, it can be assumed that patients with large ischemic lesions are at greater risk of hemorrhagic transformation. High systolic blood pressure was another clinical factor more frequently noticed in patients with sICH. Uncontrolled high blood pressure is a well-known contraindication for the administration of IVT for the treatment of acute ischemic stroke, as it has been demonstrated that it is associated with a greater risk of hemorrhagic transformation and death [11]. Several pathophysiological mechanisms have been reported to underlie this association, including endothelial cell dysfunction, oxidative stress, excessive production of inflammatory cytokines and matrix metalloproteinases (MMPs), and blood–brain barrier disruption [29]; all these mechanisms are associated with one another.

We identified several imaging markers that could be associated with sICH after ischemic stroke treated with bridging therapy. Hence, intracranial vascular calcifications on angio-CT scan and leukoaraisosis on brain MRI were more frequent in patients with sICH, which is consistent with previous studies that reported a similar association in ischemic stroke patients treated with either IVT [30] or MT [31]. Interestingly, we also found a greater prevalence of intracranial but not extracranial atheroma in patients with sICH. Although the exact mechanisms that could account for the relationship between these imaging markers and the risk of sICH are not fully understood, it can be assumed that they may reflect arterial wall fragility and damage to the blood–brain barrier, which could contribute to hemorrhagic transformation after brain reperfusion. Conversely, cerebral microbleeds were not more prevalent in patients with sICH in our study, although it has been demonstrated that they confer a greater risk of hemorrhagic transformation in ischemic stroke patients treated with IVT, especially if they are numerous [32]. Of note, the prevalence of cerebral microbleeds was surprisingly low in our cohort, so much so that no patients with sICH had microbleeds. However, only approximately two thirds of patients beneficiated from an MRI during their stay, with the same distribution between those with and without sICH. Therefore, it cannot be excluded that we missed an association between cerebral microbleeds and sICH in patients treated with bridging therapy because of unavailable data on MRI. In addition, in our MRI protocol, assessment of microbleeds was based on a T2 gradient echo (T2-GRE) sequence rather than susceptibility-weighted imaging (SWI), and it is known that the sensitivity of SWI sequencing is greater than T2-GRE sequencing for the detection of microbleeds.

Regarding treatments prior to stroke, the use of dual antiplatelet therapy was more frequent in ischemic stroke patients who had sICH. Studies that address the issue of the impact of DAPT on hemorrhagic transformation after IVT have shown conflicting results; a meta-analysis including 30,000 patients found an increased risk with an odds ratio of about 3 [33], while another including 75,000 European patients did not reveal any association [34]. Although dual antiplatelet therapy is no longer consider a contra-indication of IVT according to the current guidelines on the management of acute ischemic stroke [11], our results underline the caution that may be required with patients on this therapeutic regimen. The prevalence of premorbid use of anticoagulants did not statistically differ between the two groups, but only a few patients in our cohort were anticoagulated at the time of their stroke. This is due to the fact that IVT is contraindicated in most patients on anticoagulation therapy, and they are therefore underrepresented in the cohort of patients receiving bridging therapy compared with patients who would be treated with MT alone. Indeed, in cases of proximal occlusion, clinicians prefer MT alone to bridging in ischemic stroke patients taking anticoagulants in order to limit the risk of hemorrhagic complications.

Finally, we also evaluated several biomarkers of systemic inflammation, as inflammation has been shown to play an important role in both hemorrhagic stroke and hemorrhagic transformation of ischemic stroke by promoting blood–brain barrier disruption and the worsening of brain tissue damage [35]. We found that patients with sICH had higher neutrophil-to-lymphocyte ratios and neutrophil-to-platelet ratios. These markers have been reported to be associated with increased risk of hemorrhagic transformation in IS patients, whether or not they were treated with acute revascularization therapy [36,37,38,39].

Our study has several limits. Because of its single-center nature, our sample size was limited, and therefore we were not able to perform a multivariable analysis with sufficient power to better explore the association between studied variables and the risk of sICH, due to the risk of over-adjustment. In addition, more than one third of patients did not have an MRI during their stay, so it was not possible to exhaustively evaluate imaging markers such as cerebral microbleeds, as previously discussed. We did not administrate formal scales to assess pre-stroke cognitive status; therefore, some MCI patients may have been undiagnosed because they did not seek medical attention before their stroke or their relatives did not report cognitive problems during their interview. Finally, we cannot exclude that some unmeasured factors, such as frailty or other co-morbidities, may influence the risk of sICH after bridging therapy.

## 5. Conclusions

To conclude, this study reported that one in twelve ischemic patients treated with bridging therapy suffered sICH. Although our study was underpowered to draw any definite conclusion, we observed that these patients had more severe stroke on initial clinical examination; higher blood pressure on admission; more frequent vascular intracranial calcifications, intracranial atheroma, and leukoaraiosis on imaging; and higher blood inflammation profiles. Given the observed poor outcomes after sICH, further studies are required to better identify patients at risk, so as to help clinicians in guiding therapeutic strategies.

## Figures and Tables

**Table 1:** Baseline characteristics of ischemic stroke patients according to the occurrence of symptomatic intracerebral hemorrhage (sICH).

	Patients with sICH (n = 15)	Patients without sICH (n = 161)	*p*
***Demographics***			
Age (years old), mean ± SD	70.7 ± 8.5	68.5 ± 16.6	0.60
Male sex, n (%)	8 (53)	76 (48)	0.88
***Medical history and risk factors***			
Smoking, n (%)	7 (53.6)	76 (52.7)	0.94
Alcohol abuse, n (%)	4 (30.1)	14 (9.7)	**0.023**
BMI, median [IQR]	25.5 [22.2–29.7]	26.1 [23.5–29.3]	0.62
Hypertension, n (%)	10 (66.6)	89 (56.6)	0.45
Diabetes mellitus, n (%)	3 (20)	22 (14)	0.54
Atrial fibrillation, n (%)	4 (28.5)	49 (31.6)	0.81
Hypercholesterolemia, n (%)	4 (26.6)	40 (25.6)	0.93
Chronic kidney failure, n (%)	1 (6.6)	4 (2.5)	0.36
Chronic cardiac failure, n (%)	1 (7.1)	7 (4.7)	0.70
Current major depressive disorder, n (%)	3 (20)	17 (11.3)	0.32
History of stroke, n (%)	1 (6.6)	13 (8.4)	0.81
Sleep apnea syndrome, n (%)	3 (21.4)	15 (9.7)	0.17
Cognitive impairment, n (%)	0 (0)	17 (11.1)	0.22

SD: standard deviation; BMI: body mass index.

**Table 2:** Prior-to-stroke treatments of ischemic stroke patients according to the occurrence of symptomatic intracerebral hemorrhage (sICH).

	Patients with sICH (n = 15)	Patients without sICH (n = 161)	*p*
Aspirin alone, n (%)	1 (7.1)	26 (17.1)	0.33
Clopidogrel alone, n (%)	0 (0)	2 (1.3)	0.66
DAPT, n (%)	2 (14.3)	2 (1.3)	**0.002**
Anticoagulants, n (%)	0 (0)	10 (6.5)	0.32
Antihypertensive therapy (any), n (%)	12 (85.7)	96 (62.5)	0.08
Beta-blockers, n (%)	6 (42.8)	55 (35.4)	0.62
ACE inhibitors, n (%)	3 (21.4)	26 (17.1)	0.68
Calcic inhibitors, n (%)	4 (28.5)	22 (14.4)	0.16
Diuretics, n (%)	5 (35.7)	48 (31.5)	0.75
ARBs, n (%)	1 (7.1)	32 (21.0)	0.21
Antidiabetics, n (%)	1 (7.1)	10 (6.5)	0.93
Statin therapy, n (%)	4 (28.5)	29 (19.0)	0.39
Antidepressants, n (%)	3 (21.4)	13 (8.4)	0.11
Antipsychotics, n (%)	1 (7.1)	4 (2.6)	0.33

DAPT: dual antiplatelet therapy; ACE: angiotensin-converting enzyme; ARBs: angiotensin receptor blockers.

**Table 3:** Clinical and imaging features on admission of ischemic stroke patients according to the occurrence of symptomatic intracerebral hemorrhage (sICH).

	Patients with sICH (n = 15)	Patients without sICH (n = 161)	*p*
***Clinical features***			
Wake-up stroke, n (%)	3 (20)	17 (10.7)	0.34
IVT during telemedicine procedure, n (%)	10 (66.6)	88 (55.3)	0.39
NIHSS score on admission, median [IQR]	20 [13–24]	14.5 [8.5–19.5]	**0.01**
Altered consciousness on admission, n (%)	5 (33.3)	18 (11.4)	**0.01**
Capillary glycemia, median [IQR]	1.23 [1.16–1.39]	1.15 [0.99–1.34]	0.16
Systolic blood pressure mmHg, median [IQR]	175 [164–181]	150.5 [136–170]	**0.02**
Systolic blood pressure ≥ 160 mmHg, n (%)	7 (77.7)	39 (36.1)	**0.01**
Systolic blood pressure ≥ 150 mmHg, n (%)	7 (77.7)	56 (51.8)	0.13
Diastolic blood pressure mmHg, median [IQR]	89 [84–92]	81 [70–88]	0.053
Diastolic blood pressure ≥ 80 mmHg, n (%)	8 (88.8%)	63 (58.8%)	0.07
***Occlusion site***			0.12
ICA, n (%)	0 (0)	8 (5.1)	
Tandem or T-occlusion, n (%)	5 (33.3)	27 (17.3)	
M1 or M1-M2 junction segment of MCA, n (%)	8 (50)	90 (58.0)	
M2 segment of MCA, n (%)	2 (12.5)	10 (6.5)	
Basilar or vertebral artery, n (%)	0 (0)	21 (13.5)	
***Acute therapy procedures***			
Onset-to-needle time, median [IQR], min	140 [110–180]	164.5 [134–205]	0.36
Onset-to-groin puncture time, median [IQR], min	362 [241–456]	293 [185–392]	0.41
MT procedure duration, median [IQR], min	54 [41–90]	50 [39–69]	0.31
General anesthesia for MT, n (%)	7 (46.6)	60 (38.4)	0.53
Successful reperfusion (TICI 2b/3), n (%)	9 (75.0)	107 (82.3)	0.91
***Imaging data***			
Presence of microbleeds, n (%) *	0 (0)	8 (7.9)	0.41
Presence of vascular calcifications, n (%)	13 (86.6)	69 (47.7)	**0.004**
Intracranial atheroma, n (%)	12 (75.0)	73 (49.6)	**0.02**
Leukoaraiosis, n (%) *	10 (100)	51 (44.7)	**0.001**
Extracranial atheroma, n (%)	9 (75)	97 (66.8)	0.56
ICA stenosis, n (%)	3 (25.0)	32 (22.5)	0.84
***Stroke etiology***			
Cardio-embolic stroke, n (%)	5 (33.3)	69 (46.0)	0.34
Large artery atherosclerosis stroke, n (%)	3 (20)	27 (18.4)	0.84

* among patients who had brain MRI; IQR: interquartile range; IVT: intravenous thrombolysis; MT: mechanical thrombectomy; TICI: treatment in cerebral ischemia scale; ICA: internal carotid artery; MCA: middle cerebral artery.

**Table 4:** Biological variables of ischemic stroke patients, upon admission, according to the occurrence of symptomatic intracerebral hemorrhage (sICH).

	Patients with sICH (n = 15)	Patients without sICH (n = 161)	*p*
CRP (mg/L), median [IQR]	6.1 [3.5–25.8]	5.4 [2.9–13.4]	0.40
Leukocytes, median [IQR]	11.1 [9–14.1]	9.7 [7.8–11.8]	0.12
Platelets, median [IQR]	214.5 [193–268]	225 [187–263]	0.78
TSH, median [IQR]	0.95 [0.67–1.78]	1.08 [0.60–1.75]	0.93
HbA1c, median [IQR]	5.8 [5.6–6.0]	5.8 [5.5–6.2]	0.73
Creatinine, median [IQR]	72.5 [59–82]	71.0 [60–82]	0.76
Troponin I, mean ± SD	0.08 ± 0.16	0.17 ± 84	0.75
Monocytes/HDL, median [IQR]	0.65 [0.60–0.81]	0.54 [0.38–0.77]	0.14
Neutrophil-to-lymphocyte, median [IQR]	6.1 [3.8–10.2]	4.1 [2.5–6.9]	**0.02**
Neutrophil-to-platelet, median [IQR]	0.81 [0.71–0.87]	0.62 [0.72–0.81]	**0.04**
LDL cholesterol, median [IQR]	3.4 [3.1–3.5]	2.8 [2.1–3.6]	0.08
HDL cholesterol, median [IQR]	1.23 [1.17–1.35]	1.29 [1.04–1.51]	0.90
Total cholesterol, median [IQR]	5.15 [4.89–5.70]	4.64 [3.92–5.57]	0.07

TSH: thyroid-stimulating hormone; CRP: C-reactive protein; HbA1C: hemoglobin A1C; HDL: high-density lipoprotein; LDL: low-density lipoprotein.

**Table 5:** Rates of sICH in patients included in the bridging therapy group of randomized clinical trials aiming to compare mechanical thrombectomy with bridging therapy in patients with ischemic stroke due to large vessel occlusion.

Trial	Definition of sICH	sICH Rate in Bridging Therapy Group
DIRECT-MT [23]	Heidelberg classification	6.1%
MR CLEAN-NO IV [24]	Heidelberg classification	5.3%
DEVT [25]	Heidelberg classification	6.8%
	ECASS-II criteria	8.5%
	ECASS-III criteria	6.0%
	NINDS criteria	10.3%
	SITS-MOST criteria	4.3%
SKIP [26]	NINDS criteria	11.7%
	SITS-MOST criteria	7.8%
DIRECT-SAFE [27]	Intracerebral hemorrhage (parenchymal hematoma type 2 (PH2) within 72 h of treatment) combined with neurological deterioration leading to an increase of ≥4 points in the NIHSS score from baseline, or the lowest NIHSS value between baseline and 24 h.	1%
SWIFT-DIRECT [28]	SICH_global_: core laboratory-adjudicated parenchymal hematoma type 1 or 2, subarachnoid hemorrhage, or intraventricular hemorrhage within 24 h (±6 h), associated with an increase in the NIHSS score of 4 or more, compared with baseline.	3%
	SICH_site_: site investigator-adjudicated evidence of any intracranial hemorrhage, and site investigator-adjudicated neurological worsening of 4 points in the NIHSS score compared with immediately before deterioration, most likely due to radiologically evident intracranial hemorrhage.	5%

## Data Availability

The data presented in this study are available on request from the corresponding author. The data are not publicly available for legal reasons.
